# Supplementary material for: Generalizability is not optional: insights from a cross-cultural study of social discounting
Source: R Soc Open Sci. 2019 Feb 27;6(2):181386. doi: 10.1098/rsos.181386 (PMC6408392; doi:10.1098/rsos.181386)
Supplement: Additional characteristics of participants / responses, sensitivity checks, and exploratory analyses [file rsos181386supp1.pdf]

SUPPLEMENTAL MATERIALS FOR:

Generalizability is not optional: Insights from a cross-cultural study of social discounting.

Leonid Tiokhin*[1, 2], Joseph Hackman*[1], Shirajum Munira[3], Khaleda Jesmin[3], Daniel Hruschka*[1]

*Equal Contributions

[1] Arizona State University School of Human Evolution and Social Change

[2]Human Technology Interaction Group, Eindhoven University of Technology

[3]LAMB Project for Integrated Health and Development, Parbatipur 5250, Bangladesh

Open access data, materials, and code available at https://osf.io/cfkdr/

Correspondence concerning this article should be addressed to: Leonid Tiokhin, Human Technology Interaction Group, Eindhoven University of Technology, IPO 1.24, PO Box 513, 5600 MB, Eindhoven, the Netherlands. Email: l.tiokhin@tue.nl

CONTENTS

PARTICIPANT DEMOGRAPHIC CHARACTERISTICS 3
MODEL COMPARISON 4
Testing the Best Model for All Sites 4
Chi-Square Tests for Model Fit 6
ALTERNATIVE MODEL SPECIFICATIONS 8
All Sites 8
Within Sites 15
BIC AND BAYES FACTORS FOR MODELS WITH/WITHOUT SOCIAL DISTANCE 21
EXCLUSIONS AND INCLUSIONS 24
Excluding Participants Who Gave Nothing to All Recipients 24
Including Participant Decisions Towards "Unknown Person" 26
GENEROSITY AS A FUNCTION OF PAYOFF TO PARTICIPANT 28
INTERACTION BETWEEN PARTICIPANT CONSISTENSY AND GENEROSITY 29
BIC AND BAYES FACTORS FOR MODELS WITH/WITHOUT PARTICIPANT-CONSISTENCY INTERACTION 33
POWER ANALYSIS 35
INCONSISTENT RESPONDING ACROSS SITES 37
EXPECTED SHARING AS A MEASURE OF GENEROSITY 39
RELATIONSHIP BETWEEN EXPECTED SHARING AND CROSSOVER POINTS 40
RE-ANALYSIS USING APPROXIMATE CROSSOVER POINTS 42
CODEBOOK FOR VERBAL STATEMENTS 44
REFERENCES 46

PARTICIPANT DEMOGRAPHIC CHARACTERISTICS

	U.S.	Bangladesh	Indonesia
**N**	40	200	44
**Age (mean(s.d.))**	19.5 (1.3)	38.0 (14.3)	34.5 (9.6)
**Sex (female/male (% female))**	18/21 (46%)	166/200 (83%)	25/18 (58%)

Table S1 | Participant Demographic Characteristics. Two participants (one U.S., one Indonesian) reported their sex as "other" and were excluded from estimates of the male-female ratio.

MODEL COMPARISON

Testing the Best Model for All Sites

Model	DF	Log Likelihood	AIC	ΔAIC	BIC (Max N = 1388)	ΔBIC (Max N = 1388)	BIC (Min N = 284)	ΔBIC (Min N = 284)
Full Model (2 Ran. Slopes)	26	509.30	-966.59	0	-830.47	15.35	-871.73	8.99
No Ran. Slope Social Distance	22	502.5	-961	5.59	-845.82	0	-880.72	0
No Ran. Slope Need	19	395.97	-753.95	212.64	-654.47	191.35	-684.61	196.11
No Ran. Slope Social Distance or Need; Ran. Slope Relatedness	19	389.63	-741.27	225.32	-641.79	204.03	-671.93	208.79
Ran. Intercept Only	17	387.33	-740.65	225.94	-651.65	194.17	-678.63	202.09
No Ran. Effects	16	167.52	-303.05	663.54	-219.28	626.54	-244.66	636.06

Table S2 | Information criteria for different random-effect structures. Full Model (2 Ran. Slopes; Table 1, main text) includes fixed-effects for social distance, relatedness, and relative need, a random intercept for participant, and random slopes for both relative need and social distance. Table S2 compares this model to alternative models that differ only in their random-effects. No Ran. Slope Social Distance = random intercept for participant and random slope for need. No Ran. Slope Need = random intercept for participant and random slope for social distance. No Ran. Slope Social Distance or Need; Ran. Slope Relatedness = random intercept for participant and random slope for relatedness. Ran. Intercept only = random intercept for participant. No Ran. Effects = linear model with no random effects. 2 columns for Bayesian Information Criteria (BIC) indicate the upper and lower bounds on BIC. BIC with Max N = 1388 calculates BIC assuming each observation is independent. BIC with Min N = 284 calculates BIC assuming only 1 observation per participant (i.e. all observations for a given participant are entirely non-independent). ΔAIC and ΔBIC indicate the differences in information criteria between alternative models and the best model.

Model	DF	AIC	AIC Weight	BIC (Max N = 1388)	BIC Weight (Max N = 1388)	BIC (Min N = 284)	BIC Weight (Min N = 284)
Full Model (2 Ran. Slopes)	26	-966.59	0.94	-830.47	0	-871.73	0.01
Ran. Slope Need	22	-961	0.06	-845.82	1	-880.72	0.99
Ran. Slope Social Distance	19	-753.95	0	-654.47	0	-684.61	0
Ran. Slope Relatedness	19	-741.27	0	-641.79	0	-671.93	0
Ran. Intercept Only	17	-740.65	0	-651.65	0	-678.63	0
No Ran. Effects	16	-303.05	0	-219.28	0	-244.66	0

Table S3 | AIC and BIC Weights. Full Model (2 Ran. Slopes; Table 1, main text) includes fixed-effects for social distance, relatedness, and relative need, a random intercept for participant, and random slopes for both relative need and social distance. Table S3 compares the AIC and BIC weights of this model to alternative models that differ only in their random-effects (See Table S2 above)

Chi-Square Tests for Model Fit

Random intercept for participant is significant

Model 1: Fixed Effects (ln Social Distance, Relative Need, Relatedness); Random Effects (None)

Model 2: Fixed Effects (ln Social Distance, Relative Need, Relatedness); Random Effects (Random Intercept for Participant)

**Model**	**DF**	**ΔDF**	**Chisq**	**P-Value**
Model 1	16			
Model 2	17	1	439.61	<0.001

Random slope for social distance is significant

Model 2: Fixed Effects (ln Social Distance, Relative Need, Relatedness); Random Effects (Random Intercept for Participant)

Model 3: Fixed Effects (ln Social Distance, Relative Need, Relatedness); Random Effects (Random Intercept for Participant; Random Slope for ln Social Distance)

**Model**	**DF**	**ΔDF**	**Chisq**	**P-Value**
Model 2	17			
Model 3	19	2	17.3	<0.001

Random slope for relative need is significant

Model 2: Fixed Effects (ln Social Distance, Relative Need, Relatedness); Random Effects (Random Intercept for Participant)

Model 4: Fixed Effects (ln Social Distance, Relative Need, Relatedness); Random Effects (Random Intercept for Participant; Random Slope for Relative Need)

**Model**	**DF**	**ΔDF**	**Chisq**	**P-Value**
Model 2	17			
Model 4	22	5	230.35	<0.001

Random slope for relatedness is not significant

Model 2: Fixed Effects (ln Social Distance, Relative Need, Relatedness); Random Effects (Random Intercept for Participant)

Model 5: Fixed Effects (ln Social Distance, Relative Need, Relatedness); Random Effects (Random Intercept for Participant; Random Slope for Relatedness)

**Model**	**DF**	**ΔDF**	**Chisq**	**P-Value**
Model 2	17			
Model 5	19	2	4.62	0.10

A model with random slopes for both relative need and ln social distance is significantly better than a model with only a random slope for need or only a random slope for social distance

Model 3: Fixed Effects (ln Social Distance, Relative Need, Relatedness); Random Effects (Random Intercept for Participant; Random Slope for ln Social Distance)

Model 4: Fixed Effects (ln Social Distance, Relative Need, Relatedness); Random Effects (Random Intercept for Participant; Random Slope for Relative Need)

Model 6: Fixed Effects (ln Social Distance, Relative Need, Relatedness); Random Effects (Random Intercept for Participant; Random Slopes for ln Social Distance and Relative Need)

**Model**	**DF**	**ΔDF**	**Chisq**	**P-Value**
Model 3	19			
Model 6	26	7	226.65	<0.001

**Model**	**DF**	**ΔDF**	**Chisq**	**P-Value**
Model 4	22			
Model 6	26	4	13.60	0.009

ALTERNATIVE MODEL SPECIFICATIONS

All Sites

	U.S. Expected Sharing		Bangladesh Expected Sharing		Indonesia Expected Sharing	
	Estimate (CI)	*P*	*Estimate (CI)*	*P*	*Estimate (CI)*	*P*
**Fixed Effects**						
(Intercept)	0.70 (0.57 – 0.83)	<.001	0.15 (0.07 – 0.23)	<.001	0.66 (0.55 – 0.77)	<.001
Ln Social Distance	-0.10 (-0.12 – -0.08)	<.001	0.00 (-0.01 – 0.01)	.627	-0.00 (-0.02 – 0.01)	.620
Need						
Recipient Equally Needy	-0.10 (-0.22 – 0.03)	.140	-0.08 (-0.16 – 0.01)	.069	-0.19 (-0.29 – -0.09)	<.001
Recipient Less Needy	-0.19 (-0.32 – -0.06)	.004	-0.13 (-0.21 – -0.05)	.001	-0.30 (-0.41 – -0.19)	<.001
Relatedness	0.07 (-0.07 – 0.20)	.325	-0.01 (-0.10 – 0.08)	.866	0.13 (0.00 – 0.25)	.049

Table S4 | Generosity as a function of social distance, relative need, and relatedness. Only random slope for relative need. Multilevel model of social distance, recipient need, and relatedness regressed on expected sharing. Model controls for correlated observations from the same participant with random effects for each individual and includes a random slope for recipient need. CI = 95% confidence intervals.

	U.S. Expected Sharing		Bangladesh Expected Sharing		Indonesia Expected Sharing	
	Estimate (CI)	*P*	*Estimate (CI)*	*P*	*Estimate (CI)*	*P*
**Fixed Effects**						
(Intercept)	0.71 (0.58 – 0.84)	<.001	0.15 (0.07 – 0.22)	<.001	0.70 (0.59 – 0.80)	<.001
Ln Social Distance	-0.10 (-0.12 – -0.08)	<.001	0.00 (-0.01 – 0.01)	.661	-0.01 (-0.03 – 0.01)	.226
Need						
Recipient Equally Needy	-0.10 (-0.22 – 0.02)	.117	-0.07 (-0.15 – 0.01)	.087	-0.20 (-0.30 – -0.10)	<.001
Recipient Less Needy	-0.19 (-0.32 – -0.07)	.004	-0.13 (-0.21 – -0.05)	.001	-0.31 (-0.42 – -0.20)	<.001

Table S5 | Generosity as a function of social distance and relative need (excluding genetic relatedness). Multilevel model of social distance and recipient need regressed on expected sharing. Model controls for correlated observations from the same participant with random effects for each individual and includes random slopes for social distance and recipient need. CI = 95% confidence intervals.

	U.S. Expected Sharing		Bangladesh Expected Sharing		Indonesia Expected Sharing	
	Estimate (CI)	*P*	*Estimate (CI)*	*P*	*Estimate (CI)*	*P*
**Fixed Effects**						
(Intercept)	0.61<br>(0.54 – 0.67)	<.001	0.04<br>(0.02 – 0.07)	.003	0.52<br>(0.45 – 0.59)	<.001
Ln Social Distance	-0.11<br>(-0.13 – -0.09)	<.001	0.00<br>(-0.01 – 0.01)	.875	-0.03<br>(-0.05 – -0.01)	.013
Relatedness	0.04<br>(-0.12 – 0.19)	.653	-0.04<br>(-0.15 – 0.07)	.453	0.14<br>(0.00 – 0.27)	.046

Table S6 | Generosity as a function of social distance and relatedness (excluding need). Multilevel model of social distance and relatedness regressed on expected sharing. Model controls for correlated observations from the same participant with random effects for each individual and includes a random slope for social distance. Without controlling for need, social distance has a stronger estimated association with generosity in Indonesia. CI = 95% confidence intervals.

	U.S. Expected Sharing		Bangladesh Expected Sharing		Indonesia Expected Sharing	
	Estimate (CI)	*P*	*Estimate (CI)*	*P*	*Estimate (CI)*	*P*
**Fixed Effects**						
(Intercept)	0.61<br>(0.55 – 0.68)	<.001	0.04<br>(0.01 – 0.07)	.004	0.55<br>(0.49 – 0.61)	<.001
Ln Social Distance	-0.11<br>(-0.14 – -0.09)	<.001	0.00<br>(-0.01 – 0.01)	.775	-0.04<br>(-0.06 – -0.02)	<.001

Table S7 | Generosity as a function of social distance (excluding need and genetic relatedness). Multilevel model of social distance regressed on expected sharing. Model controls for correlated observations from the same participant with random effects for each individual and includes a random slope for social distance. When removing all co-variates, social distance has a stronger estimated association with generosity in Indonesia. CI = 95% confidence intervals.

	Expected Sharing	
	Estimate (CI)	*P*
**Fixed Effects**		
(Intercept)	0.71<br>(0.57 – 0.84)	<.001
Ln Social Distance (U.S.)	-0.10<br>(-0.12 – -0.08)	<.001
Site (Reference = U.S)		
Bangladesh	-0.56<br>(-0.71 – -0.41)	<.001
Indonesia	-0.04<br>(-0.21 – 0.13)	.670
Relative Need		
Recipient Equally Needy	-0.10<br>(-0.22 – 0.02)	.121
Recipient Less Needy	-0.19<br>(-0.32 – -0.07)	.004
Relatedness	0.05<br>(-0.08 – 0.19)	.459
Ln Social Distance: Bangladesh	0.10<br>(0.08 – 0.12)	<.001
Ln Social Distance: Indonesia	0.10<br>(0.07 – 0.12)	<.001
Bangladesh: Recipient Equally Needy	0.03<br>(-0.12 – 0.17)	.721
Indonesia: Recipient Equally Needy	-0.10<br>(-0.25 – 0.06)	.224
Bangladesh: Recipient Less Needy	0.06<br>(-0.09 – 0.21)	.401
Indonesia: Recipient Less Needy	-0.11<br>(-0.28 – 0.06)	.191

Bangladesh: Relatedness	-0.06<br>(-0.22 – 0.10)	.475
Indonesia: Relatedness	0.07<br>(-0.11 – 0.25)	.452
**Random Parts**		
σ^2	0.016	
$\tau_{00, \text{respid}}$	0.080	
ρ_{01}	-0.750	
N_{respid}	284	
Observations	1388	
R^2 / Ω_0^2	.882 / .879	

Table S8 | Generosity as a function of social distance, relative need, and relatedness (Full output for Table 1 in main text). Multilevel model of social distance, relative need, and relatedness regressed on expected sharing. Model controls for correlated observations from the same participant with random effects for each individual and includes random slopes for social distance and relative need. Model compares effect estimates in Bangladesh and Indonesia to the U.S. (i.e. the reference group). CI = 95% confidence intervals.

	U.S. Expected Sharing		Bangladesh Expected Sharing		Indonesia Expected Sharing	
	Estimate (CI)	*P*	*Estimate (CI)*	*P*	*Estimate (CI)*	*P*
**Fixed Effects**						
(Intercept)	0.68 (0.55 – 0.80)	<.001	0.15 (0.07 – 0.23)	<.001	0.67 (0.57 – 0.78)	<.001
Social Distance	-0.02 (-0.02 – -0.01)	<.001	0.00 (-0.00 – 0.00)	.618	-0.00 (-0.01 – 0.00)	.315
Need						
Recipient Equally Needy	-0.11 (-0.23 – 0.02)	.095	-0.08 (-0.16 – 0.00)	.064	-0.20 (-0.30 – -0.10)	<.001
Recipient Less Needy	-0.20 (-0.33 – -0.08)	.002	-0.13 (-0.21 – -0.06)	<.001	-0.30 (-0.41 – -0.19)	<.001
Relatedness	0.04 (-0.09 – 0.18)	.548	-0.01 (-0.10 – 0.08)	.870	0.11 (-0.01 – 0.23)	.081

Table S9 | Generosity as a function of social distance (unlogged), relative need, and relatedness. Multilevel model of raw (unlogged) social distance, relative need, and relatedness regressed on expected sharing. Model controls for correlated observations from the same participant with random effects for each individual and includes random slopes for social distance and relative need. CI = 95% confidence intervals.

Within Sites

	Bangladesh Expected Sharing	
	Estimate (CI)	*P*
**Fixed Effects**		
(Intercept)	0.13 (0.05 – 0.22)	.002
Ln Social Distance	0.00 (-0.00 – 0.01)	.444
Relative Need		
Recipient Equally Needy	-0.07 (-0.15 – 0.01)	.106
Recipient Less Needy	-0.12 (-0.20 – -0.04)	.004
Relatedness	-0.01 (-0.06 – 0.04)	.799
Order_Asked		
Order_Asked2	0.01 (-0.00 – 0.03)	.120
Order_Asked3	-0.01 (-0.02 – 0.01)	.260
Order_Asked4	-0.00 (-0.02 – 0.01)	.718
Order_Asked5	0.00 (-0.02 – 0.02)	.954
Age	0.00 (-0.00 – 0.00)	.914
Recipient Gender (Reference Category = Female)		
Male	0.00 (-0.01 – 0.01)	.911

Unspecified	0.01 (-0.16 – 0.17)	.949
Participant Gender (Reference Category = Female)	0.01 (-0.01 – 0.04)	.343
**Random Parts**		
σ^2	0.005	
$\tau_{00, \text{respid}}$	0.088	
ρ_{01}	0.077	
N_{respid}	200	
Observations	964	
R^2 / Ω_0^2	.876 / .874	

Table S10 | Generosity among Bangladesh participants as a function of social distance, relative need, and relatedness, controlling for participant and recipient gender, order of recipient, and participant age. Multilevel model of social distance, recipient need, and relatedness regressed on expected sharing. Model also includes fixed effects for participant gender, recipient gender, order or recipient, and participant age. Model controls for correlated observations from the same participant with random effects for each individual and includes random slopes for social distance and recipient need. CI = 95% confidence intervals.

	Indonesia Expected Sharing	
	Estimate (CI)	*P*
**Fixed Effects**		
(Intercept)	0.69 (0.46 – 0.93)	<.001
Ln Social Distance	-0.00 (-0.03 – 0.03)	.871
Relative Need		
Recipient Equally Needy	-0.23 (-0.33 – -0.13)	<.001
Recipient Less Needy	-0.34 (-0.46 – -0.22)	<.001
Relatedness	0.12 (-0.07 – 0.31)	.210
Order_Asked		
Order_Asked2	0.03 (-0.06 – 0.12)	.464
Order_Asked3	-0.01 (-0.10 – 0.08)	.780
Order_Asked4	0.03 (-0.07 – 0.12)	.589
Order_Asked5	-0.02 (-0.12 – 0.07)	.623
Order_Asked6	0.08 (-0.19 – 0.36)	.569
Age	0.00 (-0.01 – 0.01)	.783
Recipient Gender (Reference Category = Female)		
Male	-0.03 (-0.11 – 0.04)	.402

Unspecified	0.06 (-0.10 – 0.21)	.464
Participant Gender (Reference Category = Female)	-0.05 (-0.17 – 0.07)	.436
**Random Parts**		
σ^2	0.039	
$\tau_{00, respid}$	0.027	
ρ_{01}	-0.273	
N_{respid}	43	
Observations	215	
R^2 / Ω_0^2	.737 / .722	

Table S11 | Generosity among Indonesian participants as a function of social distance, relative need, and relatedness, controlling for participant and recipient gender, order of recipient, and participant age. Multilevel model of social distance, recipient need, and relatedness regressed on expected sharing. Model also includes fixed effects for participant gender, recipient gender, order of recipient, and participant age. Model controls for correlated observations from the same participant with random effects for each individual and includes a random slope for recipient need. CI = 95% confidence intervals.

	U.S. Expected Sharing	
	Estimate (CI)	*P*
**Fixed Effects**		
(Intercept)	0.58 (-0.68 – 1.84)	.376
Ln Social Distance	-0.11 (-0.14 – -0.08)	<.001
Relative Need		
Recipient Equally Needy	-0.13 (-0.24 – -0.01)	.032
Recipient Less Needy	-0.22 (-0.33 – -0.11)	<.001
Relatedness	0.14 (-0.09 – 0.37)	.223
Order_Asked		
Order_Asked2	-0.13 (-0.23 – -0.02)	.020
Order_Asked3	-0.08 (-0.19 – 0.03)	.145
Order_Asked4	-0.20 (-0.31 – -0.10)	<.001
Order_Asked5	-0.11 (-0.21 – -0.00)	.051
Order_Asked6	0.01 (-0.10 – 0.12)	.808
Age	0.01 (-0.05 – 0.08)	.729
Recipient Male (Reference Category = Female)	0.04 (-0.03 – 0.12)	.284

Participant Male (Reference Category = Female)	0.02 (-0.15 – 0.19)	.842
**Random Parts**		
σ^2	0.047	
$\tau_{00, \text{respid}}$	0.061	
N_{respid}	39	
Observations	195	
R^2 / Ω_0^2	.728 / .722	

Table S12 | Generosity among U.S. participants as a function of social distance, relative need, and relatedness, controlling for participant and recipient gender, order, and participant age. Multilevel model of social distance, recipient need, and relatedness regressed on expected sharing. Model also includes fixed effects for participant gender, recipient gender, order of recipient, and participant age. Model controls for correlated observations from the same participant with random effects for each individual. CI = 95% confidence intervals.

BIC AND BAYES FACTORS FOR MODELS WITH/WITHOUT SOCIAL DISTANCE

We calculate Bayesian Information Criterion (BIC)[1] values to assess the extent to which the data favor models (i.e. statistical descriptions of hypotheses) with or without social distance. We then use BIC values to approximate Bayes Factors (BF) for competing models[2].

**Bangladesh**	**DF**	**Log Likelihood**	**BIC (Max N = 968)**	**BIC (Min N = 200)**
Full Model (2 Ran. Slopes)	16	902.25	-1694.5	-1719.73
No Ran. Slope Social Distance	12	881.47	-1680.4	-1699.36
No Ran. Slope or Fixed Effect Social Distance	11	881.06	-1686.5	-1703.84
**Indonesia**	**DF**	**Log Likelihood**	**BIC (Max N = 220)**	**BIC (Min N = 44)**
Full Model (2 Ran. Slopes)	16	-6.31	98.92	73.16
No Ran. Slope Social Distance	12	-8.02	80.77	61.45
No Ran. Slope or Fixed Effect Social Distance	11	-8.08	75.49	57.78
**U.S.**	**DF**	**Log Likelihood**	**BIC (Max N = 200)**	**BIC (Min N = 40)**
Full Model (2 Ran. Slopes)	16	-25.34	135.45	109.7
No Ran. Slope Social Distance	12	-26.74	117.05	97.75
No Ran. Slope or Fixed Effect Social Distance	11	-46.28	150.85	133.14

Table S13 | BIC for competing models. Full Model (2 Ran. Slopes; Table 1, main text) includes fixed-effects for social distance, relatedness, and relative need, a random intercept for participant, and random slopes for both relative need and social distance. This table compares this model to alternative models that differ by removing just the random slope for social distance (No Ran. Slope Social Distance) or by removing both the random and fixed effect of social distance (No Ran. Slope or Fixed Effect Social Distance). 2 columns for Bayesian Information Criteria (BIC) indicate the upper and lower bounds on BIC. BIC with Max N calculates BIC assuming each observation is independent. BIC with Min N calculates BIC assuming only 1 observation per participant (i.e. all observations for a given participant are entirely non-independent).

We approximate Bayes Factors (BF) by exponentiating half the difference between the BIC values of competing models (i.e. $\exp(\Delta BIC_{10} / 2)$).[2] BF_{10} indicates a ratio: the likelihood of the data conditional on Model 1, $P(D|M_1)$, divided by the likelihood of the data conditional on Model 0, $P(D|M_0)$. For example, if $BF_{10} = 8$, the data are 8 times more likely under Model 1 than Model 0. If $BF_{10} = 0.01$, the data are 100 times less likely under Model 1 than Model 0. For all below comparisons, Model 1 is listed first and Model 0 is listed second.

Bangladesh BF

Full Model (2 Ran. Slopes) vs. No Ran. Slope Social Distance

Using BIC Max N. $BF_{10} = 1152.86$

Using BIC Min N. $BF_{10} = 26462.93$

Full Model (2 Ran. Slopes) vs. No Ran. Slope or Fixed Effect Social Distance

Using BIC Max N. $BF_{10} = 54.60$

Using BIC Min N. $BF_{10} = 2818.61$

No Ran. Slope Social Distance vs. No Ran. Slope or Fixed Effect Social Distance

Using BIC Max N. $BF_{10} = 0.05$

Using BIC Min N. $BF_{10} = 0.11$

Indonesia BF

Full Model (2 Ran. Slopes) vs. No Ran. Slope Social Distance

Using BIC Max N. $BF_{10} = 0.0001$

Using BIC Min N. $BF_{10} = 0.0029$

Full Model (2 Ran. Slopes) vs. No Ran. Slope or Fixed Effect Social Distance

Using BIC Max N. $BF_{10} = 0.000008$

Using BIC Min N. $BF_{10} = 0.00046$

No Ran. Slope Social Distance vs. No Ran. Slope or Fixed Effect Social Distance

Using BIC Max N. $BF_{10} = 0.07$

Using BIC Min N. $BF_{10} = 0.16$

U.S. BF

Full Model (2 Ran. Slopes) vs. No Ran. Slope Social Distance

Using BIC Max N. $BF_{10} = 0.0001$

Using BIC Min N. $BF_{10} = 0.0026$

Full Model (2 Ran. Slopes) vs. No Ran. Slope or Fixed Effect Social Distance

Using BIC Max N. $BF_{10} =$ 2208.35

Using BIC Min N. $BF_{10} =$ 123007.4

No Ran. Slope Social Distance vs. No Ran. Slope or Fixed Effect Social Distance

Using BIC Max N. $BF_{10} =$ 21856305

Using BIC Min N. $BF_{10} =$ 48399498

In Bangladesh and Indonesia, BF indicate support for a model without a fixed effect for social distance, whereas in the U.S., BF indicate support for a model with a fixed effect for social distance. In Indonesia and the U.S., BF also indicate support for a model without a random slope for social distance, whereas in Bangladesh, BF indicate support for a model with a random slope for social distance.

EXCLUSIONS AND INCLUSIONS

Excluding Participants Who Gave Nothing to All Recipients

	U.S. Expected Sharing		Bangladesh Expected Sharing		Indonesia Expected Sharing	
	Estimate (CI)	*P*	*Estimate (CI)*	*P*	*Estimate (CI)*	*P*
**Fixed Effects**						
(Intercept)	0.74 (0.59 – 0.89)	<.001	0.41 (0.28 – 0.55)	<.001	0.69 (0.56 – 0.82)	<.001
Ln Social Distance	-0.10 (-0.14 – -0.07)	<.001	0.01 (-0.03 – 0.04)	.615	-0.01 (-0.04 – 0.03)	.715
Need						
Recipient Equally Needy	-0.12 (-0.26 – 0.03)	.118	-0.15 (-0.30 – -0.00)	.052	-0.20 (-0.32 – -0.08)	.001
Recipient Less Needy	-0.21 (-0.36 – -0.06)	.007	-0.30 (-0.43 – -0.16)	<.001	-0.31 (-0.44 – -0.18)	<.001
Relatedness	0.05 (-0.16 – 0.26)	.630	-0.03 (-0.36 – 0.31)	.878	0.13 (-0.07 – 0.32)	.202
**Random Parts**						
σ^2	0.042					
$\tau_{00, respid}$	0.077					
ρ_{01}	-0.692					
N_{respid}	116					
Observations	576					
R^2 / Ω_0^2	.781 / .768				.	

Table S14 | Generosity as a function of social distance, relative need, and relatedness, only including participants with non-zero generosity. Multilevel model of social distance, recipient need, and relatedness regressed on expected sharing. Model controls for correlated observations from the same participant with random effects for each individual and includes random slopes for social distance and recipient need. When excluding participants who gave nothing to all recipients, the effect of social distance on

generosity remains largely unchanged in each site. Number of participants = 35 (Bangladesh), 39 (U.S.), 42 (Indonesia). CI = 95% confidence intervals.

Including Participant Decisions Towards "Unknown Person"

To assess individual decisions unfamiliar partners, participants in all sites also made decisions between selfish and generous options for an "unknown person". Below, we reanalyze the data, including generosity towards an "unknown person".

	U.S. Expected Sharing		Bangladesh Expected Sharing		Indonesia Expected Sharing	
	Estimate (CI)	*P*	*Estimate (CI)*	*P*	*Estimate (CI)*	*P*
**Fixed Effects**						
Intercept for "Unknown Individual"	0.39 (0.28, 0.50)	<.001	0.08 (0.03, 0.13)	.003	0.60 (0.51, 0.70)	<.001
Change in Intercept for Recipients with a Social Distance	0.36 (0.30, 0.42)	<.001	0.01 (-0.02, 0.04)	.381	0.03 (-0.03, 0.09)	.291
Ln Social Distance	-0.10 (-0.12, -0.08)	<.001	0.00 (-0.01, 0.01)	.605	-0.00 (-0.02, 0.02)	.943
Need						
Recipient Equally Needy	-0.15 (-0.26, -0.05)	.003	-0.03 (-0.08, 0.02)	.257	-0.16 (-0.25, -0.08)	<.001
Recipient Less Needy	-0.24 (-0.34, -0.13)	<.001	-0.07 (-0.13, -0.02)	.006	-0.30 (-0.39, -0.21)	<.001
Relatedness	0.06 (-0.07, 0.19)	.335	-0.02 (-0.10, 0.07)	.722	0.16 (0.04, 0.28)	.010
**Random Parts**						
σ^2	0.018					
$\tau_{00, \text{respid}}$	0.057					
ρ_{01}	0.086					
N_{respid}	284					
Observations	1671					
R^2 / Ω_0^2	.858 / .855					

Table S15 | Generosity as a function of social distance, relative need, and relatedness, including data for generosity towards an "unknown person". Multilevel model of social distance, recipient need, relatedness, and whether recipient had a social-distance ranking (categorical) regressed on expected sharing. Model controls for correlated observations from the same participant with random effects for each individual and includes random slopes for social distance and recipient need. 'Intercept for "Unknown Individual"' indicates the estimate for participant generosity towards an "unknown individual". 'Change in Intercept for Recipients with a Social Distance" indicates the change in intercept, relative to 'Intercept for "Unknown Individual"', for recipients who have a social-distance ranking (i.e. the expected sharing towards a recipient at social distance = 1). Number of participants = 200 (Bangladesh), 40 (U.S.), 44 (Indonesia). CI = 95% confidence intervals.

GENEROSITY AS A FUNCTION OF PAYOFF TO PARTICIPANT

	U.S. Odds of Sharing		Bangladesh Odds of Sharing		Indonesia Odds of Sharing	
	Estimate (CI)	*P*	*Estimate (CI)*	*P*	*Estimate (CI)*	*P*
**Fixed Effects**						
(Intercept)	0.17 (0.08, 0.38)	<.001	0.00 (0.00, 0.00)	<.001	0.47 (0.29, 0.78)	.003
Decision						
2	2.62 (1.51, 4.55)	<.001	1.56 (0.81, 3.02)	.184	2.27 (1.47, 3.50)	<.001
3	6.76 (3.86, 11.84)	<.001	1.73 (0.90, 3.32)	.101	3.33 (2.15, 5.16)	<.001
4	16.88 (9.39, 30.36)	<.001	3.65 (1.94, 6.86)	<.001	2.88 (1.86, 4.45)	<.001
5	39.17 (20.81, 73.71)	<.001	7.10 (3.80, 13.26)	<.001	2.74 (1.78, 4.24)	<.001
6	88.42 (43.73, 178.78)	<.001	12.61 (6.72, 23.67)	<.001	3.50 (2.25, 5.43)	<.001
**Random Parts**						
$\tau_{00, respid}$	4.503		85.830		1.713	
N_{respid}	40		200		44	
Observations	1200		5808		1320	
Deviance	908.292		876.467		1417.457	

Table S16 | Generosity as a function of payoff to participant. Logistic regression of payoff to participant (i.e. cost of sharing) regressed on sharing (binomial yes/no outcome). Larger numbers for “Decision” indicate smaller participant payoffs (i.e. smaller costs to sharing). Model controls for correlated observations from the same participant with random effects for each individual. Excludes data for generosity towards “unknown person” and “acquaintance”. In all sites, participants have higher odds of sharing on decisions when the personal costs of doing so are low.

INTERACTION BETWEEN PARTICIPANT CONSISTENSY AND GENEROSITY

	U.S. Expected Sharing	
	Estimate (CI)	*P*
**Fixed Effects**		
(Intercept)	0.74 (0.52, 0.96)	<.001
Ln Social Distance	-0.10 (-0.16, -0.03)	.004
Consistent Participant	0.03 (-0.19, 0.25)	.778
Need		
Recipient Equally Needy	-0.13 (-0.25, -0.01)	.036
Recipient Less Needy	-0.22 (-0.33, -0.11)	<.001
Relatedness	0.08 (-0.15, 0.31)	.482
Ln Social Distance * Consistent Participant Interaction	-0.01 (-0.09, 0.06)	.687
**Random Parts**		
σ^2	0.055	
$\tau_{00, respid}$	0.055	
N_{respid}	39	
Observations	195	
R^2 / Ω_0^2	.677 / .669	

Table S17 | Generosity among U.S. participants as a function of social distance, relative need, relatedness, and participant consistency, only including participants with non-zero generosity. Multilevel model of social distance, recipient need, relatedness, and participant consistency (categorical: 1, 0) regressed on expected sharing. Participants were considered inconsistent if they had multiple crossover points for at least 1 recipient. Model controls for correlated observations from the same participant with random effects for each individual and includes random slopes for social distance and recipient need. CI = 95% confidence intervals.

	Bangladesh Expected Sharing	
	Estimate (CI)	*P*
**Fixed Effects**		
(Intercept)	0.40 (0.24, 0.56)	<.001
Ln Social Distance	0.01 (-0.02, 0.05)	.420
Consistent Participant	0.07 (-0.10, 0.25)	.424
Need		
Recipient Equally Needy	-0.16 (-0.34, 0.03)	.106
Recipient Less Needy	-0.30 (-0.47, -0.14)	.002
Relatedness	-0.03 (-0.34, 0.28)	.847
Ln Social Distance * Consistent Participant Interaction	-0.01 (-0.08, 0.06)	.760
**Random Parts**		
σ^2	0.035	
$\tau_{00, \text{respid}}$	0.108	
ρ_{01}	-0.767	
N_{respid}	35	
Observations	171	
R^2 / Ω_0^2	.782 / .774	

Table S18 | Generosity among Bangladesh participants as a function of social distance, relative need, relatedness, and participant consistency, only including participants with non-zero generosity. Multilevel model of social distance, recipient need, relatedness, and participant consistency (categorical: 1, 0) regressed on expected

sharing. Participants were considered inconsistent if they had multiple crossover points for at least 1 recipient. Model controls for correlated observations from the same participant with random effects for each individual and includes a random slope for recipient need. CI = 95% confidence intervals.

BIC AND BAYES FACTORS FOR MODELS WITH/WITHOUT PARTICIPANT-CONSISTENCY INTERACTION

We calculate Bayesian Information Criterion (BIC)[1] values to assess the extent to which the data favor models (i.e. statistical descriptions of hypotheses) with or without an interaction between Ln Social Distance and Participant Consistency in Bangladesh and the U.S.. We then use BIC values to approximate Bayes Factors (BF) for competing models[2].

**U.S.**	**DF**	**Log Likelihood**	**BIC (Max N = 195)**	**BIC (Min N = 39)**
Interaction Model (Ran. Intercept)	9	-28.12	98.59	85.73
No-Interaction Model (Ran. Intercept)	8	-28.21	103.71	89.21
**Bangladesh**	**DF**	**Log Likelihood**	**BIC (Max N = 171)**	**BIC (Min N = 35)**
Interaction Model (Ran. Intercept + 1 Ran. Slope)	14	1.52	68.86	46.65
No-Interaction Model (Ran. Intercept + 1 Ran. Slope)	13	1.56	63.81	43.18

Table S19 | BIC for competing models. In the U.S., Interaction Model (Ran. Intercept) includes fixed-effects for social distance, relatedness, relative need, and participant consistency (categorical; 1, 0), an interaction between participant consistency and social distance, and a random intercept for participant. In Bangladesh, Interaction Model (Ran. Intercept + 1 Ran. Slope) is an identical model, but also includes a random slope for recipient need. Both models only include data from participants with non-zero generosity. In both sites, the No-Interaction model removes the interaction between participant consistency and social distance. Participants were considered inconsistent if they had multiple crossover points for at least 1 recipient. 2 columns for Bayesian Information Criteria (BIC) indicate the upper and lower bounds on BIC. BIC with Max N calculates BIC assuming each observation is independent. BIC with Min N calculates BIC assuming only 1 observation per participant (i.e. all observations for a given participant are entirely non-independent).

We approximate Bayes Factors (BF) by exponentiating half the difference between the BIC values of competing models (i.e. $\exp(\Delta BIC_{10} / 2)$).[2] BF_{10} indicates a ratio: the likelihood of the data conditional on Model 1, $P(D|M_1)$, divided by the likelihood of the data conditional on Model 0, $P(D|M_0)$. For example, if $BF_{10} = 8$, the data are 8 times more likely under Model 1 than Model 0. If $BF_{10} = 0.01$, the data are 100 times less likely under Model 1 than Model 0. For all below comparisons, Model 1 is listed first and Model 0 is listed second.

U.S. BF

No-Interaction Model (Ran. Intercept) vs Interaction Model (Ran. Intercept)

Using BIC Max N. $BF_{10} = 12.94$

Using BIC Min N. $BF_{10} = 5.70$

Bangladesh BF

No-Interaction Model (Ran. Intercept + 1 Ran. Slope) vs Interaction Model (Ran. Intercept + 1 Ran. Slope)

Using BIC Max N. $BF_{10} = 12.49$

Using BIC Min N. $BF_{10} = 5.67$

In both Bangladesh and the U.S., BF indicate support for a model without an interaction between social distance and participant consistency.

POWER ANALYSIS

Power of the current study to detect a significant independent effect of Social Distance on Expected Sharing, as a function of effect size. Analysis uses the powerSim() function from the SIMR[3] package in R[4]. For each simulation, powerSim() simulates new values for Expected Sharing from the model in Table 1 (main text), refits this model using those values, and performs a two-sized z-test on the simulated data. Analysis based on 1000 simulations ($\alpha = 0.05$).

Bangladesh

Effect Size	Power	95% CI
0.005	19.50%	(17.09, 22.09)
0.0075	37.00%	(34.00, 40.08)
0.010	56.30%	(53.16, 59.40)
0.012	76.10%	(73.33, 78.71)
0.015	88.80%	(86.68, 90.69)
**0.018**	**95.90%**	**(94.48, 97.04)**
0.020	99.30%	(98.56, 99.72)

Indonesia

Effect Size	Power	95% CI
0.005	7.40%	(5.85, 9.20)
0.010	15.10%	(12.94, 17.47)
0.015	30.30%	(27.46, 33.25)
0.020	46.20%	(43.08, 49.35)
0.025	64.80%	(61.75, 67.76)
0.030	81.40%	(78.85, 83.77)
0.032	84.00%	(81.58, 86.22)
0.034	86.90%	(84.65, 88.93)
0.036	91.70%	(89.81, 93.34)
0.038	93.90%	(92.23, 95.30)
**0.040**	**96.10%**	**(94.71, 97.21)**

Power of the current study to detect a significant independent effect of Social Distance on Expected Sharing, as a function of varying sample sizes. Analysis uses the powerCurve() function from the SIMR package in R, which runs powerSim() over a range of sample sizes. This allows estimation of the number of participants necessary to have sufficient power to detect an effect of the size estimated in the model. All analyses use the same specifications described for powerSim() above.

Indonesia

Power to detect an independent effect [β = -0.006] of social distance on generosity from the model in Table 1 (main text).

Sample Size	Power	95% CI
44	7.20%	(5.68, 8.98)
1144	45.40%	(42.28, 48.55)
2224	72.60%	(69.72, 75.34)
3344	88.10%	(85.93, 90.04)
**4224**	**95.00%**	**(93.46, 96.27)**

Bangladesh

Power to detect an independent effect [β = 0.002] of social distance on generosity from the model in Table 1 (main text).

Sample Size	Power	95% CI
200	11.30%	(9.40, 13.43)
1000	49.50%	(46.36, 52.65)
1800	77.00%	(74.26, 79.58)
2600	89.40%	(87.32, 91.24)
2800	90.00%	(89.05, 92.70)
**3200**	**94.70%**	**(93.12, 96.01)**

INCONSISTENT RESPONDING ACROSS SITES

We found high rates of inconsistency (i.e. multiple crossover points for at least 1 recipient) among participants in both Bangladesh and Indonesia. When considering all participants and social distances (i.e. #1, #2, #5, #10, and #20), 28 out of 200 Bangladesh participants, 42 out of 44 Indonesia participants, and 9 out of 40 U.S. were inconsistent. This underestimates rates of inconsistency in Bangladesh, since 165 out of 200 participants always chose the selfish option and were considered consistent as a result. When considering participants with non-zero generosity (i.e. those who chose the generous option at least once for at least one recipient at social distances 1 to 20), 80% Bangladesh participants (28/35) and 100% of Indonesia participants (42/42) had at least 1 inconsistent response, compared to only 26% of U.S. participants (9/39). These are strikingly high levels of inconsistency. Figure S1 plots levels of inconsistency in these 3 sites alongside all reported inconsistency rates in social-discounting studies citing Rachlin and Jones' seminal paper [5] and using a comparable protocol (data and inclusion criteria: https://osf.io/k8sbg/). In contrast to U.S. participants and the vast majority of previous studies, inconsistency is the norm among the participants from rural Indonesia and Bangladesh.

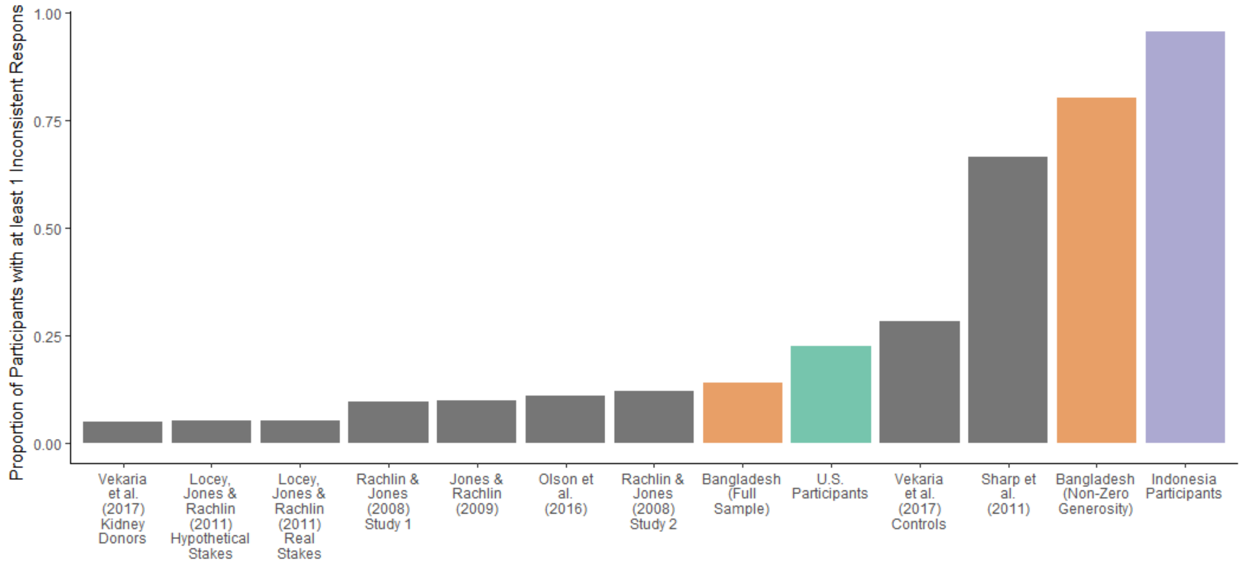

Figure S1| Inconsistent responding. Proportion of inconsistent participants (i.e. multiple crossover points for at least 1 recipient) in prior social-discounting research, compared with levels of inconsistency in the U.S., Bangladesh, and Indonesia samples from the current study. Participants from Bangladesh are represented twice (including/excluding participants with non-zero generosity).

Several observations contradict the standard interpretation that "inconsistency" reflects lack of participant understanding. First, responses in all sites were associated with a theoretically important variable—whether the recipient was needier than the participant. Second, participants were more likely to choose the generous option when the payoff for the selfish option was small (See "Generosity as a Function of Payoff to Participant" in Supplementary Materials). Third,

observations during piloting suggested that participants may not make (1) independent decisions based on (2) a constant utility function. For example, Bangladeshi participants often spoke out loud when making decisions. In such cases, many participants mentioned their decisions in previous choices while weighing current choices (e.g. “Well, I didn’t give up 1kg in the last decision, so I’ll give up 2kg this time.”; “I already gave up 5kg and 2kg rice, so I won’t give up 3kg this time”). This suggests that participants treated these as aggregate contributions, rather than as independent decisions. From this perspective, “inconsistent” responding with multiple crossover points is completely reasonable, and suggests the common model used to interpret consistent responding is wrong, at least in some situations.

EXPECTED SHARING AS A MEASURE OF GENEROSITY

"Expected sharing" is the weighted sum of all generous decisions divided by the weighted sum of all possible decisions (I.e. 0.5*X0 + 1*X1 + 2*X2 + 3*X3 + 4*X4 + 5*X5)/15.5. Here Xi = 1 if a participant sacrificed i units to give person 5 units. X0 indicates a sacrifice of 0.5 units. Although not identical to crossover points, expected sharing is monotonically increasing, provides a simple measure of average generosity towards a specific individual, and does not force exclusion of inconsistent respondents. Consider a participant who chooses to transfer $5 to the recipient, keeps $4, $3, $2, for themselves, and also chooses to transfer $1 and $0.50 to the recipient. In this case, expected sharing is simply (5 + 1 + 0.5) / (5 + 4 + 3 + 2 + 1 + 0.5) = 0.419.

RELATIONSHIP BETWEEN EXPECTED SHARING AND CROSSOVER POINTS

Typical analyses calculate the "crossover point" in the sequence of questions where respondents switch from the selfish option to the generous option[5]. Consider a consistent participant who chooses the selfish option at $5 and $4 but switches to the generous option for subsequent decisions (i.e. $3, $2, $1, $0.50). This participant's crossover point is $3.50. A participant who always chooses the generous option (i.e. chooses the generous option at $5, $4, $3, $2, $1, $0.50) is considered to have a crossover point of $5.50. A participant who always chooses the selfish option (i.e. chooses the selfish option $5, $4, $3, $2, $1, $0.50) is considered to have a crossover point of $0.25. Any given crossover point corresponds to an exact expected sharing value. For our study, these values are:

Crossover Point	Expected Sharing
0.25	0
0.75	0.5 / 15.5
2.5	1.5 / 15.5
2.5	3.5 / 15.5
3.5	6.5 / 15.5
4.5	10.5 / 15.5
5.5	1

To approximate crossover points from expected sharing values for all participants, we fit a quadratic function to these values, of the form y = 0.0001669 + 0.0149876x + 0.0302354x^2. We then used the approx() function in R[4] (https://stat.ethz.ch/R-manual/R-devel/library/stats/html/approxfun.html) to perform linear interpolation, calculating approximate crossover points for every expected sharing value. Figure S2 (below) below plots the relationship between expected sharing and crossover points.

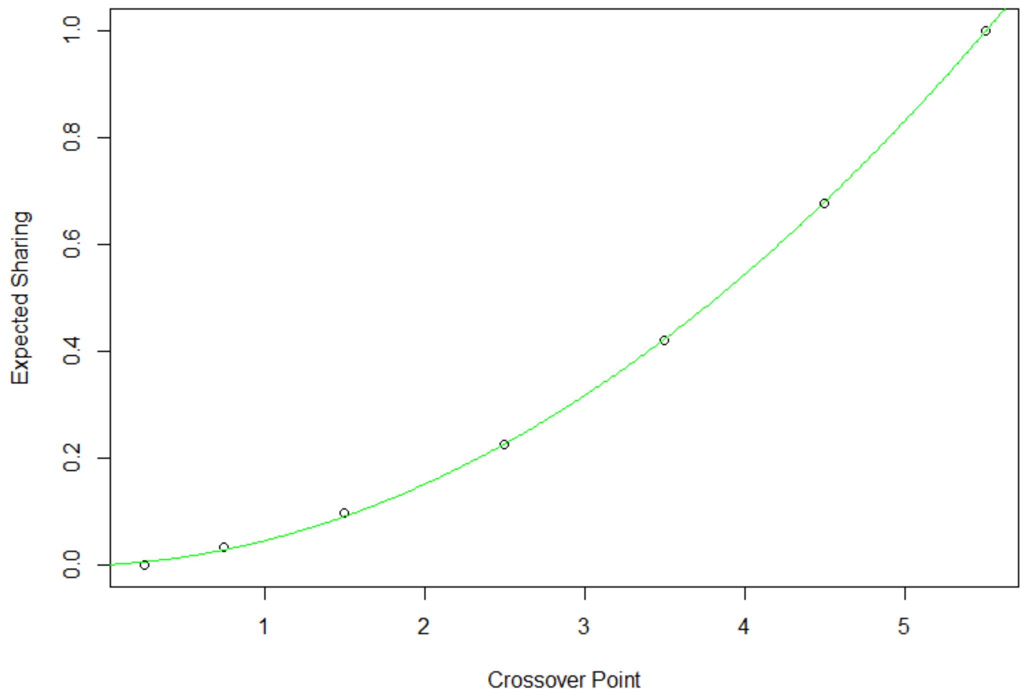

Figure S2 | Relationship between Expected Sharing and Crossover Points.

RE-ANALYSIS USING APPROXIMATE CROSSOVER POINTS

	U.S. Crossover Points		Bangladesh Crossover Points		Indonesia Crossover Points	
	Estimate (CI)	*P*	*Estimate (CI)*	*P*	*Estimate (CI)*	*P*
**Fixed Effects**						
(Intercept)	4.32 (3.64, 4.99)	<.001	1.16 (0.76, 1.55)	<.001	4.22 (3.65, 4.79)	<.001
Ln Social Distance	-0.55 (-0.66, -0.44)	<.001	0.01 (-0.04, 0.06)	.775	-0.03 (-0.15, 0.08)	.545
Need						
Recipient Equally Needy	-0.29 (-0.91, 0.33)	.370	-0.36 (-0.77, 0.05)	.090	-0.77 (-1.27, -0.28)	.003
Recipient Less Needy	-0.80 (-1.45, -0.15)	.018	-0.75 (-1.15, -0.36)	<.001	-1.45 (-2.01, -0.89)	<.001
Relatedness	0.44 (-0.28, 1.16)	.232	-0.07 (-0.55, 0.40)	.759	0.60 (-0.04, 1.25)	.066
**Random Parts**						
σ^2	0.403					
$\tau_{00, respid}$	2.192					
ρ_{01}	-0.696					
N_{respid}	284					
Observations	1388					
R^2 / Ω_0^2	.916 / .914				.	

Table S20 | Generosity as a function of social distance, relative need, and relatedness, using approximated crossover points instead of expected sharing. Multilevel model of social distance, recipient need, and relatedness regressed on approximate crossover points. Model controls for correlated observations from the same participant with random effects for each individual and includes random slopes for social distance and recipient need. CI = 95% confidence intervals.

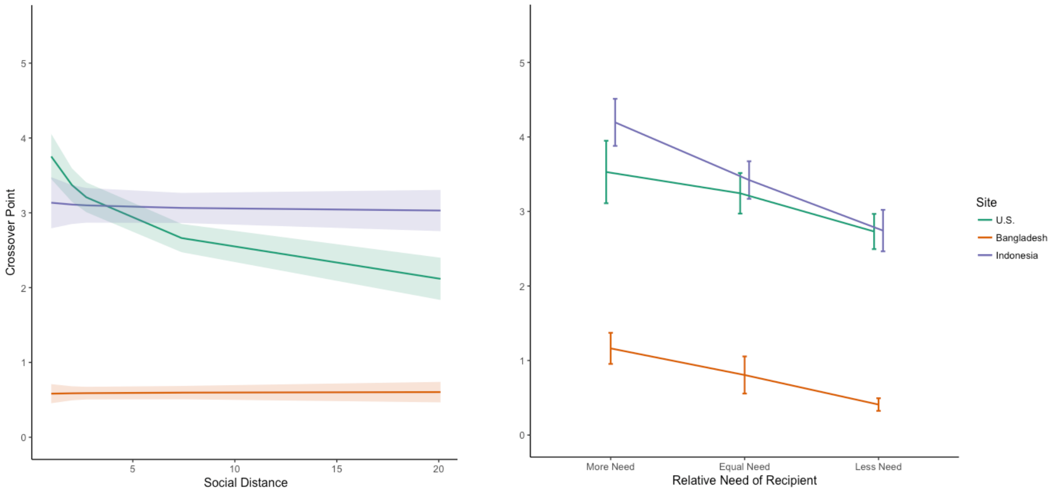

Figure S3 | Generosity as a function of social distance, relative need, and relatedness, using approximated crossover points instead of expected sharing. Independent effects of **a.** social distance (natural log transformed) and **b.** relative need on generosity (i.e. approximated crossover points). Model estimates from the multilevel model in Table 17S. Error bars represent 95% confidence intervals.

CODEBOOK FOR VERBAL STATEMENTS

Need. Any mention of the respondent's or recipient's need, financial situation, or wealth as a reason for the decision, with implication that either the respondent or recipient is in greater need. Includes mentions of general statements such as "it is good to help people who need money" and includes mentions of giving to someone who is in a needy situation (e.g. unemployed; widowed). Also includes mentions of pitying the recipient.

Relationship. Any mention of closeness, love, staying in touch, family, length of relationship or being in a relationship (e.g. good friend) as a reason for the decision. (Example: "He is my brother, but he is better off than me" would be coded as relationship, in addition to need). Includes mentions of living near the person and mentions of not knowing the person as a reason.

> **Relationship Exclusions:** If the relationship term is simply used as a description of the person (Example: "my brother is better off than me" would not be coded as relationship). If the only mention of the relationship is when respondent states that the recipient will later use the money on them or that the respondent owes the recipient. If the only reason for mentioning relationship is not knowing the other person (Example: "This person is a stranger" or "I don't know this person"), without an implication that the respondent made their decision because they did not know this person, this would not be coded as "relationship".

Reciprocity / Imbalance. Mentions giving because the other person has given to them in the past, giving in order to have the other person give to them in the future, mentions "establishing a reciprocal relationship" as a good thing, or mentions "taking turns". Also includes references to the fact that the respondent owes the recipient or that the recipient has already received too much from the respondent.

Moral. Reasoning that it is good to help others.

Descriptive. Simply states what they did (e.g., I gave to myself) without any clear reason given.

Efficiency. Refers to the fact that the respondent would give up less than the other person got.

Make Happy. Refers to the feeling that might be invoked in the recipient by the gift (happy, excited, etc.). Includes instances where respondents mentioned that the recipient would "appreciate" the gift.

Indirect Benefit. If the respondent states that the recipient will later use the money on them.

Give to something else. The respondent will keep the money to give to something or somebody else later or do something for the recipient later.

Previous decision. Respondent describes earlier decision as a justification for a later decision (I already sacrificed, so I kept for myself later; I already kept for myself, so I decided to give).

Want the money. Respondent simply stated they wanted the money.

Deserving. Respondent or recipient deserves or doesn't deserve it for reasons other than need (e.g. person is hard working, profligate)

Other. Anything that doesn't fall into the other categories (e.g. mentions of the appropriateness of an action, mentions of religion, etc.). Reasoning that it is good to help others

REFERENCES

1. Schwarz, G. Estimating the Dimension of a Model. *Ann. Stat.* **6**, 461–464 (1978).
2. Wagenmakers, E.-J. A Practical Solution to the Pervasive Problems of P Values. *Psychon. Bull. Rev.* **14**, 779 (2007).
3. Green, P. & MacLeod, C. J. SIMR: an R package for power analysis of generalized linear mixed models by simulation. *Methods Ecol. Evol.* **7**, 493–498 (2016).
4. R Core Team. R: A language and environment for statistical computing. (2017). Available at: https://www.R-project.org/.
5. Jones, B. & Rachlin, H. Social discounting. *Psychol. Sci.* **17**, 283–286 (2006).
